# InSAR constraints on soil moisture evolution after the March 2015 extreme precipitation event in Chile

**DOI:** 10.1038/s41598-017-05123-4

**Published:** 2017-07-07

**Authors:** C. P. Scott, R. B. Lohman, T. E. Jordan

**Affiliations:** 1 Department of Earth and Atmospheric Sciences, Cornell University, Ithaca, NY 14853 USA; 2 School of Earth and Space Exploration, Arizona State University, Tempe, AZ 85287 USA

## Abstract

Constraints on soil moisture can guide agricultural practices, act as input into weather, flooding and climate models and inform water resource policies. Space-based interferometric synthetic aperture radar (InSAR) observations provide near-global coverage, even in the presence of clouds, of proxies for soil moisture derived from the amplitude and phase content of radar imagery. We describe results from a 1.5 year-long InSAR time series spanning the March, 2015 extreme precipitation event in the hyperarid Atacama desert of Chile, constraining the immediate increase in soil moisture and drying out over the following months, as well as the response to a later, smaller precipitation event. The inferred temporal evolution of soil moisture is remarkably consistent between independent, overlapping SAR tracks covering a region ~100 km in extent. The unusually large rain event, combined with the extensive spatial and temporal coverage of the SAR dataset, present an unprecedented opportunity to image the time-evolution of soil characteristics over different surface types. Constraints on the timescale of shallow water storage after precipitation events are increasingly valuable as global water resources continue to be stretched to their limits and communities continue to develop in flood-prone areas.

## Introduction

InSAR^1, 2^ has contributed to our understanding of land surface change on a variety of fronts, including constraints on displacements associated with the seismic cycle^3–5^, anthropogenic activity^6–10^, landslides^11^, and volcanic unrest^12–15^. Spatial coherence of the interferometric phase^16^ is a function of the position and stability of individual scatterers within a given resolution element, typically with dimensions of meters to tens of meters. For most surface types, radar interacts with scatterers within a layer with finite thickness^17^, varying from a few cm up to 10’s of meters for vegetation, ice, and dry sand^18–20^. Variations in coherence due to imaging geometry and ground characteristics are associated with a range of phenomena of societal and scientific interest, including infrastructure damage during destructive events^21, 22^, vegetation structure and soil moisture. Constraints on vegetation structure can be derived from both air- or spaceborne SAR amplitude, phase and/or phase coherence, particularly when data is available from multiple viewing geometries^23^, wavelengths^24^, and/or polarizations^25, 26^.

The dependence of radar backscatter amplitude on soil moisture has long been recognized^17, 27–29^, as have effects on phase and phase coherence that are thought to be due to a combination of swelling and buckling of the surface and changes in the relative strength of SAR scattering centers distributed within each pixel^30–33^. These effects are closely linked to variations in the dielectric properties of the soil^33–36^ – in drier soils the radar interacts with a larger depth range and there is more contribution to the backscattered signal from scatterers at larger depths below the surface than there is in soils with higher moisture content. Because the strength of individual scatterers within a resolution element changes, interferograms between dates with different moisture levels are less coherent than those with similar moisture content.

**Figure 1:**
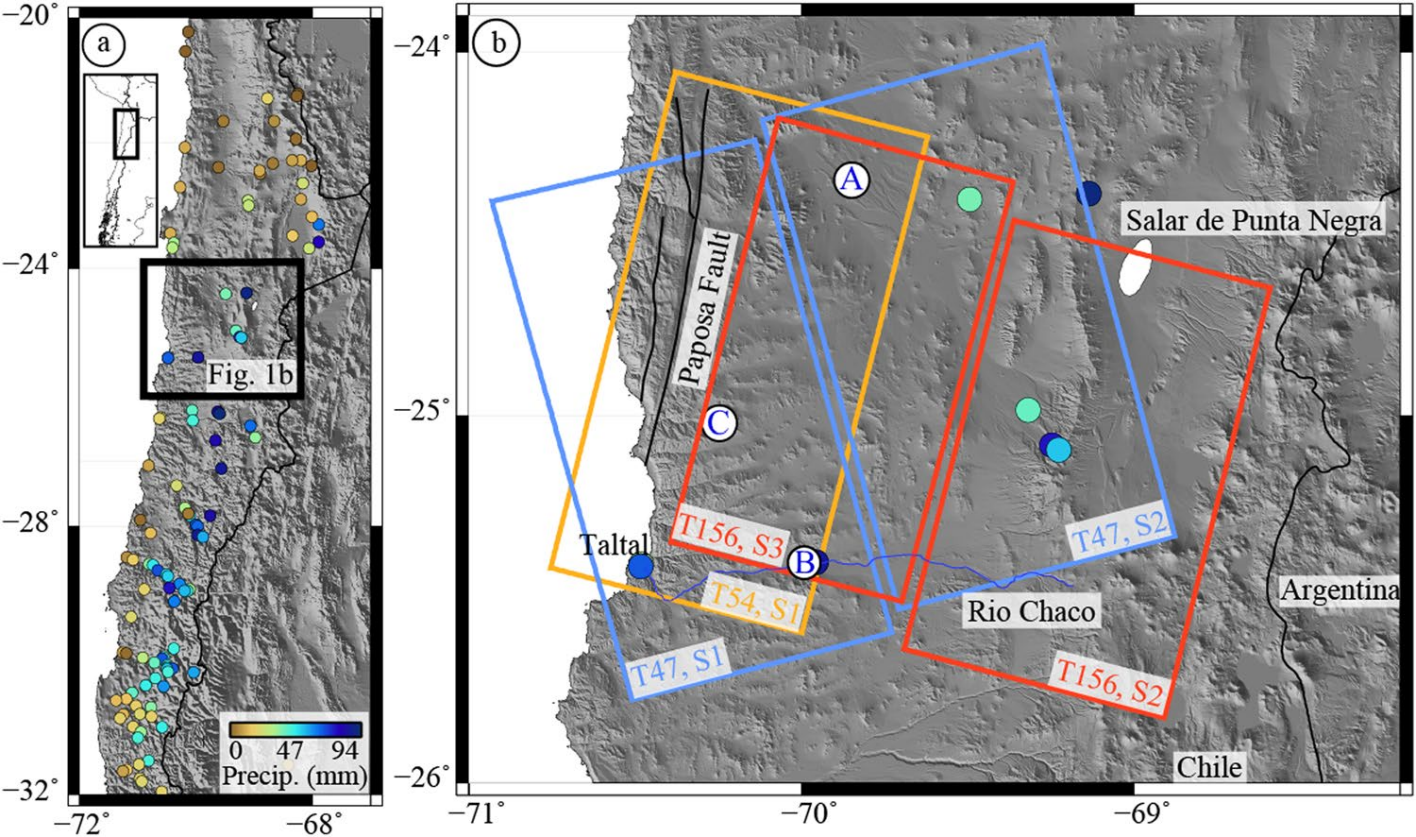
Study location and March 2015 precipitation. (**a**) Extent of the extreme precipitation event as measured at rain gauges (colored dots), overlain on shaded relief. (**b**) Location of Sentinel-1A imagery used here (boxes with track (T) and swath (S) indicated, colored by track), city of Taltal, and locations of time series shown in Figs 3 and 4 (white dots with letters, A: alluvial fan, B: along Rio Chaco, C: location in Coastal Cordillera). Figures generated using Generic Mapping Tools^50^, v. 5.2.1.

The SAR dataset used here spans an extreme rain event on 24–26 March, 2015, that covered >400,000 km^2^ of arid lands in Chile and Argentina^37–39^ and deposited up to decades-worth of the mean annual precipitation within a few days (Fig. 1a). In some river valleys there was catastrophic damage to property and loss of life, whereas other regions with similar precipitation amounts exhibited little to no surface disturbance^37–39^. Field reconnaissance efforts in the months following the event^40^ evaluated the potential for groundwater recharge through analysis of soil moisture in pits at selected sites and at a few time intervals, but were unable to sample the full variability across the region. In much of the study area, much smaller amounts of rain were also recorded in August 2015. Because the region has little to no vegetation, we are able to examine how the interferometric phase characteristics between SAR images vary over time and attribute much of the variation to temporal variations in soil properties.

## Results

**Figure 2:**
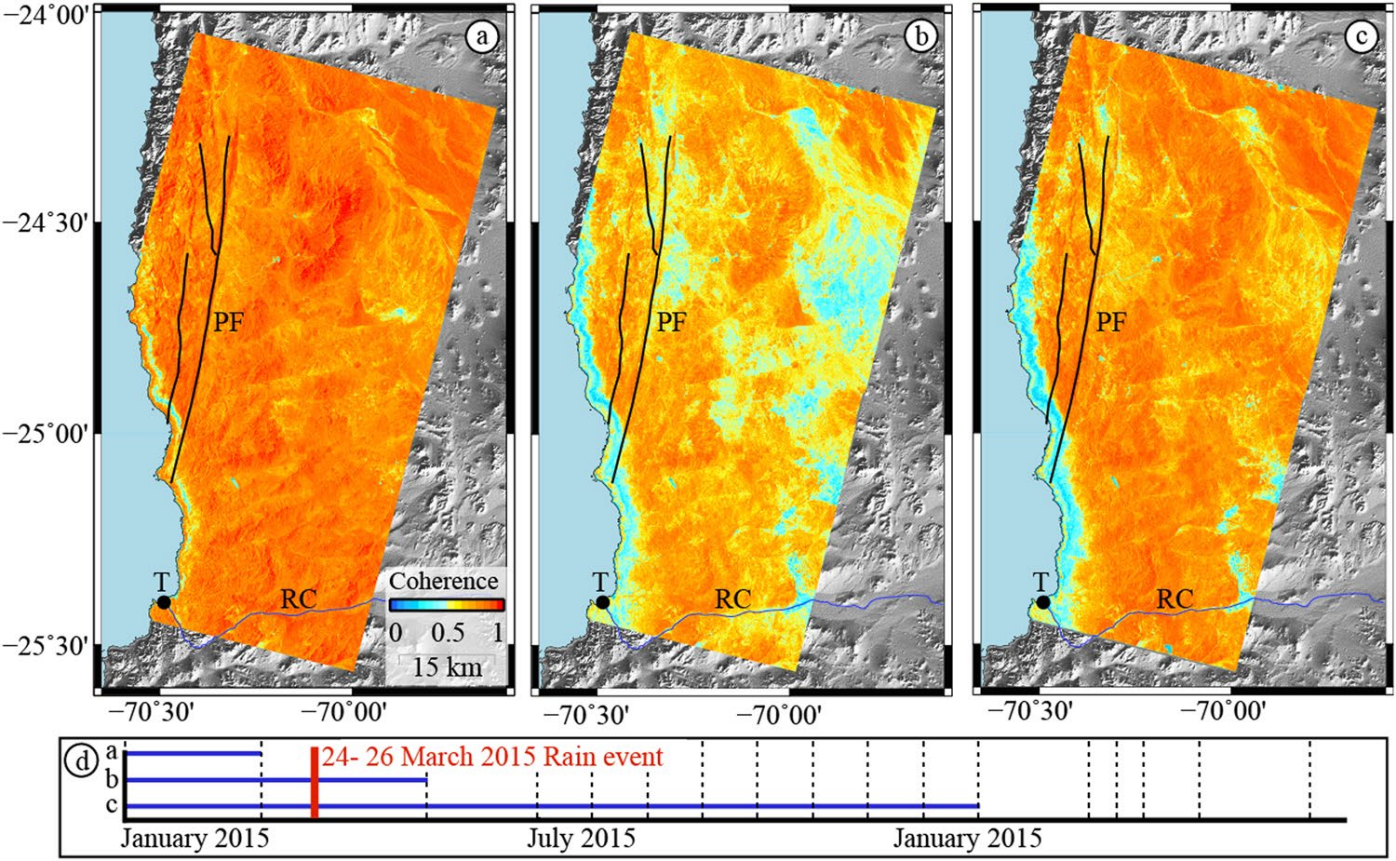
Coherence of three interferograms from Track 54, Swath 1. Interferograms between January 1, 2015, and 3 different dates: (**a**) before the event - March 2, 2015, (**b**) soon after the event - May 13, 2015, (**c**) significantly after the event - January 18, 2016. Note that the year-long interferogram has higher coherence than the 4.5 month interferogram, except for in regions with significant relief, such as the coastal escarpment north of Taltal. (**d**) Timespan of interferograms shown in **a**–**c** (blue bars), relative to timing of 24–26 March rain event (red bar). T: City of Taltal, RC: Rio Chaco, PF: Paposa Fault. All available dates shown in Supplementary Figure 1. Figures generated using Generic Mapping Tools^50^, v. 5.2.1.

We form interferograms between all pairs of available dates in three Sentinel-1A tracks (see Methods and Supplementary Tables 1–3), covering a 100 km × 150 km area located where the March 2015 precipitation was particularly heavy (Fig. 1b), and examine the phase coherence as a function of time. We show that the effects on coherence from other sources (timespan, orbital geometry) can be separated from those that are due to a proxy for soil moisture, for a result that is consistent between independent, overlapping tracks. Typically, interferograms that span longer time intervals typically have lower coherence than interferograms with shorter time spans. However, for this dataset, the least coherent interferograms are those that include the dates in the months immediately after the rain event, regardless of timescale. Even interferograms with very long time spans (>1 year) between two “dry” dates have much higher coherence than any of those that include dates immediately after the rain event (Fig. 2).

**Figure 3:**
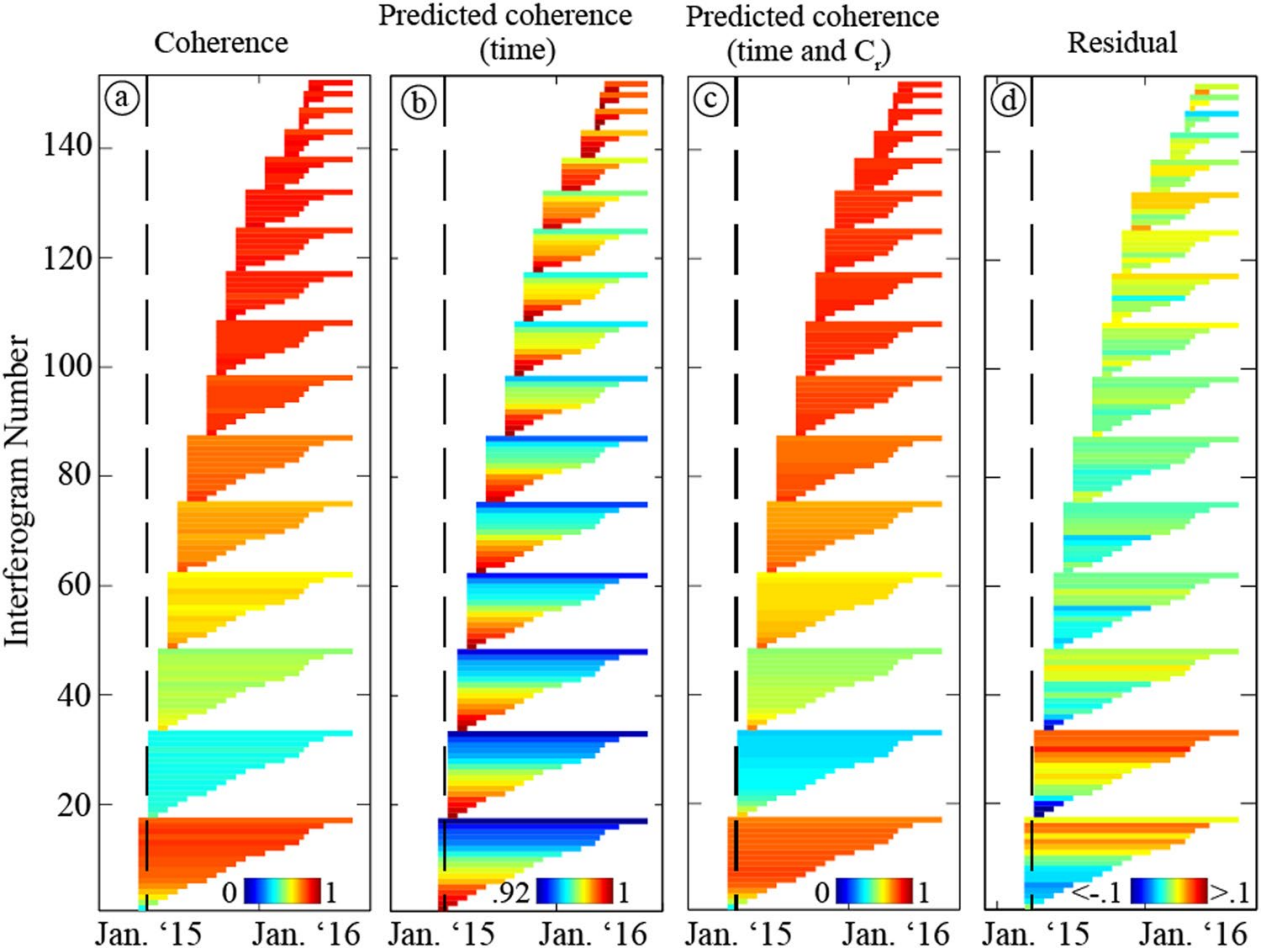
Full coherence dataset at an alluvial fan site (Track 156). (**a**) Interferometric coherence between all available acquisitions for Track 156 (Supplementary Table 1, Supplementary Figure 2), sorted by master, then secondary date, and colored by coherence at alluvial fan point (A) indicated in Fig. 1b. Vertical black line indicates timing of March 2015 rain event. (**b**) Expected coherence due to timespan alone, based only on SAR data acquired after October 1, 2015. (**c**) Coherence predicted from timespan and proxy for soil moisture. (**d**) Residual between (**a**) and (**c**). Note smaller color scale in **b** and **d**.

Each of the three independent tracks we examine here contains 11–14 dates acquired at least six months after the event, allowing us to capture the full temporal evolution from dates that are clearly affected by the rain event to those where coherence behavior appears to have returned to “normal”. The interferograms (Supplementary Tables 1–3) have a wide range of spatial and temporal baselines, both of which adversely affect coherence as they become larger^16^. We examine the decrease of coherence as a function of time and baseline in order to verify that the changes in the immediate aftermath of the rain event are not associated with those variables. At each pixel, we solve for the decrease in coherence as a function of temporal and spatial baseline using only interferograms between the subset of dates that are at least six months after the event (Fig. 3b, see Methods). We find that the dependence on spatial baseline is negligible (i.e., not significantly different from zero) except for over some higher-relief areas, which we hereafter omit from consideration in our conclusions. The dependence on temporal baseline results in decreases of less than 0.1 (coherence ranges from 0 to 1) except for along the coastal escarpment and other high relief areas that are not a focus of this study.

We attribute the remaining variation in coherence to changes in soil moisture following the rain events as well as permanent loss of coherence due to surface disruption and sediment transport. The former effect results in lower coherence only in interferograms that include dates immediately after the rain event (i.e., interferograms with time intervals of six months or more spanning the event have high coherence), while the latter results in lower coherence for any interferogram that spans the event. Over most of the study region interferograms between dates before the March rain event and dates long afterwards (six months or more) are also very coherent, indicating that the loss of coherence was “temporary” and was associated with variations in soil moisture. However, at other locations, interferograms between the pre-event and the later, post-event dates are consistently lower. We interpret this effect as a permanent loss of coherence, C_p_.

**Figure 4:**
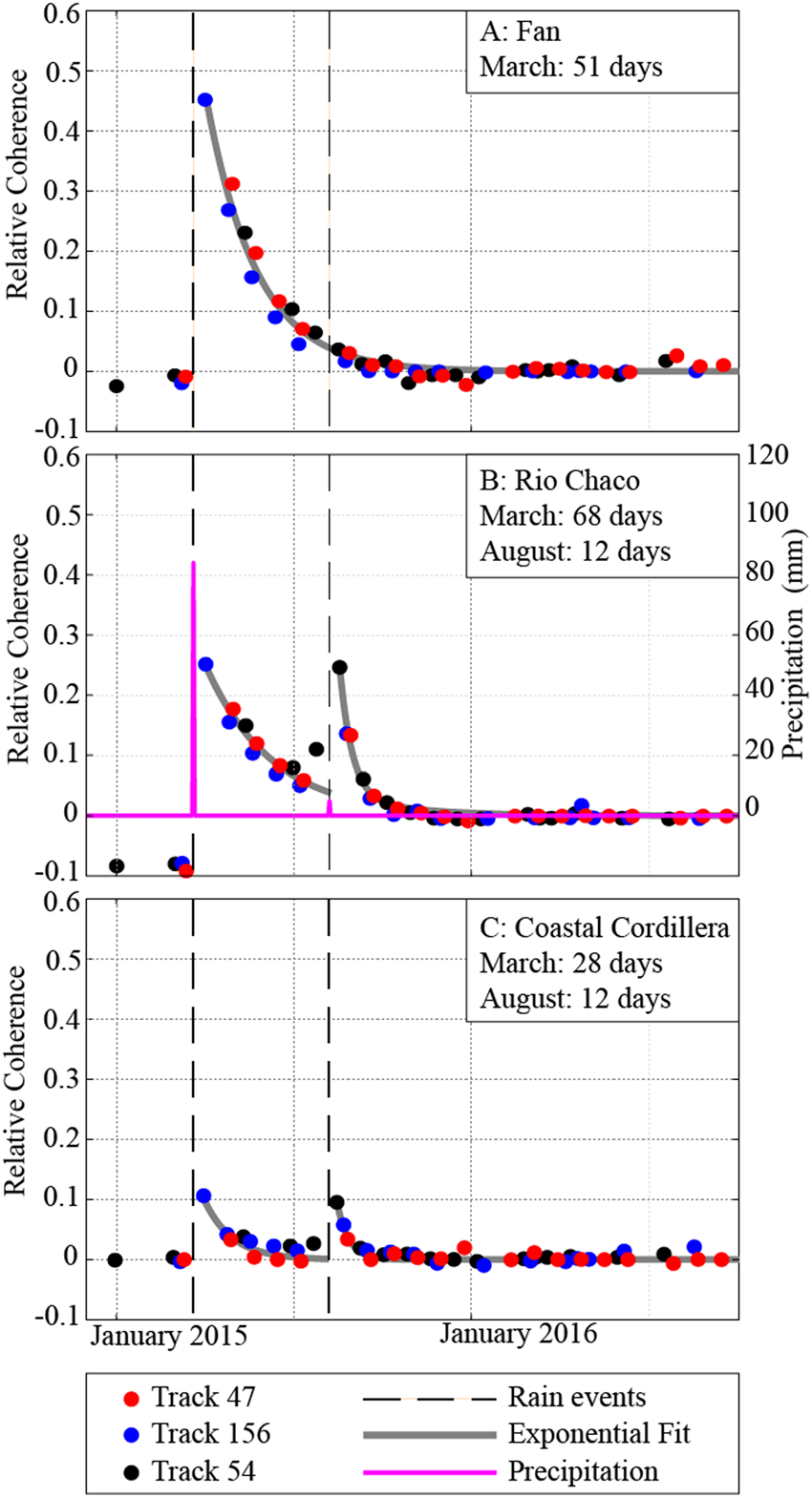
Relative coherence as a function of time. Time-series results for three independent tracks (colored dots) and the best-fit exponential decay for the larger (March) and smaller (August) rain events with locations of pixels shown as A, B, and C in Fig. 1b. (**A**) Alluvial fan site also shown in Fig. 3. (**B**) River valley site, where rain gauge observations (pink) indicate a second rain event. (**C**) Site within the coastal cordillera. Evidence of the 2^nd^ rain event can be seen, but both rain events have smaller magnitude.

At each pixel, we invert for the “relative coherence”, C_r_, at every date such that the predicted coherence of the interferogram between each pair of dates (after correction for temporal decay) depends on the absolute value of the difference in C_r_ at those dates (Fig. 4, Supplemental Figures 3–5, see Methods). The functional form of the relationship between coherence and differences in soil moisture should depend on the absolute amount of soil moisture as well as the difference in moisture between the two SAR acquisition dates^19, 31, 33, 36^. However, our approach captures most of the variability in the coherence signal (Fig. 3c), including the months-long temporal decay following the rain event. (Fig. 4). The poorest fits to the data are for the first dates acquired immediately after the rain event for each track (Fig. 3d). The change in relative coherence can be fit with an exponential decay with time constants of ~50 days (Fig. 4). The second rain event in August of 2015 is associated at some locations (Fig. 4b) with a second pulse of relative coherence variation with a similar amplitude but shorter decay time than that observed for the March event. The interferograms do not robustly support inversion for a second permanent loss of coherence during the second event, but there is likely some permanent impact that does occur below the noise level in our data.

**Figure 5:**
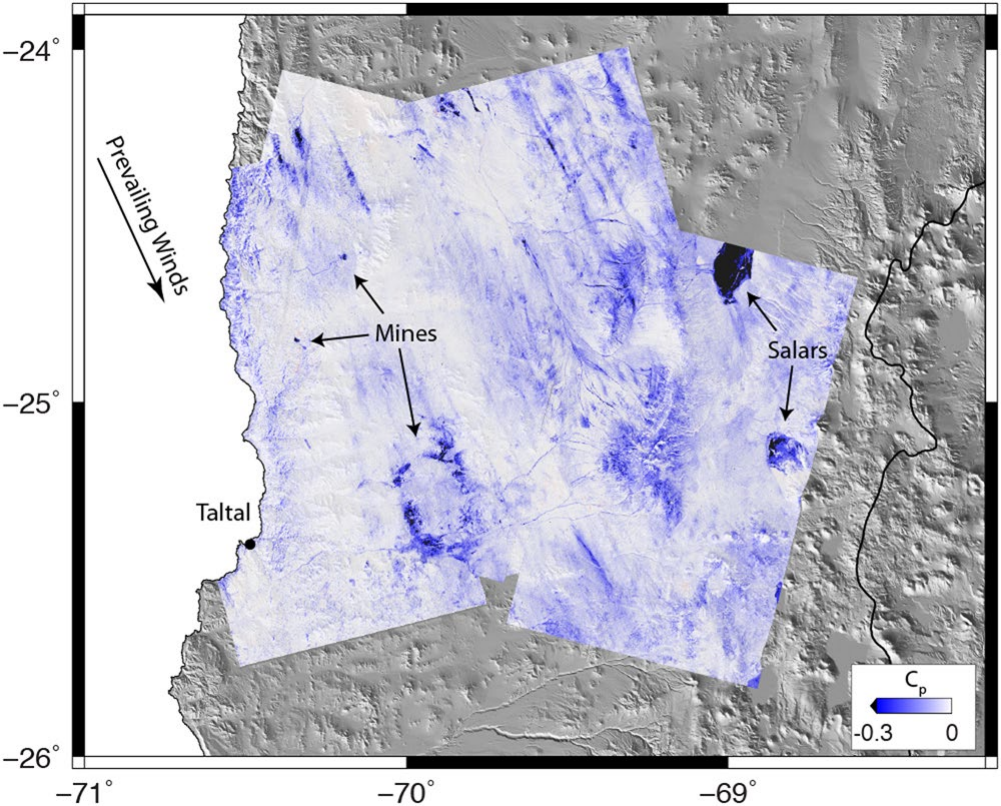
Permanent coherence loss, C_p_. Permanent coherence loss between dates before and after the March extreme precipitation event, C_p_, for all three tracks used in this study. Negative values (blue) indicate regions where land was disturbed during the event, values <−0.3 are in black. Dry lake beds (salars) and mines were particularly affected. Note that the NNW-trending streaks, which otherwise might be attributed to ionospheric effects on a particular date, are continuous from track to track and are nearly identical in the areas of overlap. These likely indicate real surface disturbance and are aligned with the prevailing winds active during the March event^37, 38^. Figures generated using Generic Mapping Tools^50^, v. 5.2.1.

In regions that are covered by multiple, independent tracks of data (including imaging from different flight directions), the time series of relative coherence is remarkably consistent between tracks (Fig. 4). Coherence between dates that were both acquired before the March rain event is high (Track 54, Fig. 2a, black dots in Fig. 4). Permanent losses of coherence, C_p_, associated with the rain event are apparent in all tracks and agree in regions of overlap. The largest losses of coherence appear to be due to sediment transport in river channels (Fig. 4b) and at sites that had been disturbed by mining (Fig. 5). Other sites (Fig. 4a,c) do not show any appreciable permanent loss of coherence. Prominent NNW-trending streaks in C_p_ (Fig. 5) are consistent with inferred prevailing wind patterns during the storm^41^ and suggest a high degree of heterogeneity in land surface disturbance.

## Discussion

The rich set of satellite-based SAR imagery spanning this event illustrates the potential of dense InSAR time series for use in soil moisture research. We image both the coherence loss associated with land disturbance during the event and its immediate aftermath, as well as a months-long perturbation to soil moisture over the region. Such observations, particularly when combined with field measurements or other remote sensing data (such as variations in SAR amplitude), are valuable input into efforts to understand the budget of infiltration and evaporation that impact soil moisture and groundwater recharge in arid environments.

The region examined here is hyperarid, with little to no vegetation. In areas with vegetation, particularly where crops are grown, we would expect the coherence behavior to have a large contribution from properties of the vegetation and their temporal changes^26, 34, 42, 43^. However, even if we restrict ourselves to arid regions, there are many interesting problems that can be addressed with the rich, openly available catalogs of SAR data that are now becoming available through the Sentinel-1 and other satellite remote sensing acquisition missions. For instance, there has been an outstanding question of whether variations in coherence and phase such as those we see here are due to swelling of clays and soils at the surface, or due to changes in the strength of the scatterers distributed beneath the surface under changing moisture conditions^30, 31, 33, 44^. Our data set contains at least one observation that may shed light on this question. At rain gauge locations where the second, August, event is detected (pink line in Fig. 4b), the observed precipitation was much smaller in magnitude than it was in March. Interestingly, the peak magnitude of our inferred C_r_ variation is similar, while the temporal decay is shorter for the second event. If the variation in coherence is due to swelling of clays, our observations require that the soils somehow “unswell” to the exact same state they were in before, so that longer time interval interferograms that span the event are associated with high coherence. Additionally, we may expect the clays at the immediate Earth surface to dry out at a fairly uniform and fast rate, particularly in such a hyperarid environment, rather than the longer and nonuniform rates that we observed. The change in rates may be explained to some degree by seasonality, since the two events occurred in late summer (March) and winter (August), respectively.

On the other hand, if the coherence is related to the integration of radar backscatter within a layer with finite depth (on order of one to a few wavelengths), then alternatives exist which may better fit the observations. For example, alluvial fans where the moisture content of the soil was increased significantly by the rain, capillary action may move moisture upward toward the surface layer, replenishing evaporated near-surface moisture; this may continue over many weeks. Another example may exist in the regions whose surface is dominated by calcium sulfate, where there is likely a cycle in which rain water that infiltrated the highly porous soil will initially partially dissolve the mineral salts which form pore walls, and then during evaporation precipitate new calcium sulfate crystals. This dissolution-precipitation cycle changes the physical state of materials in the upper tens of centimeters below the surface. By either of those mechanisms, the smaller event would change the soil moisture at very shallow depths by a similar amount as did the larger event, but the lack of deeper penetration of moisture into the subsurface would result in a shorter time scale for return to the original conditions.

Apart from the potential for characterization of hydrology and surface characteristics, InSAR observations, particularly those from modern satellite platforms with regular repeat times and open access to data, are also increasingly valuable for rapid characterization of damage associated with events such as earthquakes and large storms^21, 22, 45, 46^. While the computational requirements are high, our results suggest that analysis of background variations in coherence associated with storms or seasonal effects may improve such forecasts. This may be particularly true in arid environments where variations in moisture, rather than infrastructure damage, may dominate the change in correlation over time. This work suggests that ongoing, multi-year SAR observations of arid and hyperarid regions (including coherence, phase delays and amplitude) and the development of a catalog of precipitation events can provide constraints on the temporal evolution of soil moisture and support future field or laboratory efforts.

## Methods

### InSAR data and processing

We use data acquired by the European Space Agency’s Sentinel-1A satellite in the Terrain Observation by Progressive Scans (TOPS) mode (run in the interferometric wide swath mode) for three independent, overlapping tracks (Supplementary Tables 1–3), for the subswaths that cover our study region. Images from a single track do not image exactly the same area on each pass, resulting in some pixels that are in one subswath on some dates and the adjacent subswath on other dates. We only perform our analysis on pixels that are located within the same subswath on all dates, resulting in a small gap between the subswaths that can be seen in Fig. 1b. We process all potential interferometric pairs with data acquired between January 2015 and September 2016 using the InSAR Scientific Computing Software (ISCE) package^47^. We produce between 153 and 190 interferograms for each of the three tracks, including up to two of the available swaths for each track (Fig. 1b). Most of the images were acquired in VV polarization, but we see no variation between those and images acquired in VV + HV.

### Interferometric Coherence

Interferometric coherence^16^ is a measure of the similarity in the reflective ground properties at the timing of two SAR acquisitions, s_1_ and s_2_. The complex-valued correlation function (γ) is typically defined as:

1 $$\gamma =\frac{\langle {s}_{1}{s}_{2}^{\ast }\rangle }{\sqrt{\langle {s}_{1}{s}_{1}^{\ast }\rangle \langle {s}_{2}{s}_{2}^{\ast }\rangle }}$$

where s_i_ is the complex-valued SAR backscattered field (magnitude and phase) recorded for each acquisition. The ensemble averages 〈⋅〉 are typically evaluated by averaging in space over some number of pixels, N. We estimate γ over a box with dimensions of 7 pixels in the range direction and 3 pixels in the azimuth direction. Full resolution pixels from the Sentinel mission in the interferometric wide swath mode have a ground dimension of approximately 5 meters in range and 20 meters in azimuth. In our results shown here we do not use the amplitude weighting in Equation 1, since we find that the natural, unvegetated terrain in this research area contains amplitude variations that are completely uncorrelated with the variance of the phase (i.e., dark, smooth surfaces adjacent to bright, rough surfaces with identical phase stability). We are primarily interested in the temporal behavior of phase stability in a region where the temporal decorrelation is nearly zero – i.e., where surface roughness is not changing over time. Surface roughness strongly affects coherence and backscattered amplitude^48^, and would need to be examined in order to derive proxies for soil moisture in an absolute sense. Below, we refer to coherence as C = abs(γ), which will have low values (near 0.3) for regions that have experienced significant ground disturbance and high value (near 1) for regions that have not experienced much change in the position or characteristics of scatterers.

The small box size used in our estimate of phase variance allows us to capture sharp spatial gradients and steps between different lithologies. However, small box sizes have been shown to bias the estimate of coherence slightly upwards, particularly for low values^19, 49^. Our focus is on the temporal evolution of coherence, and focuses primarily on high coherence regions where the expected bias is small (<0.04 in correlation units for our least coherent pairs). We repeated the analysis described below at several locations using the correction for effective averaging over N = 9 and N = 25 and with and without amplitude-weighting (Supplementary Figure 6), but found no significant variation in the temporal evolution of relative coherence.

Products derived from these values (described below) are generated in the slant-range coordinate system for each track (i.e., not terrain corrected) and then the final products are georeferenced before comparison between different tracks.

### Coherence modeling

Processes occurring before, during, and after the rain event are all expected to reduce interferometric coherence, C at a particular pixel, i, and between times t_1_ and t_2_:

2 $$C(i,{t}_{1}{t}_{2})=1-{C}_{0}(i)-\frac{{\rm{\Delta }}t}{\tau (i)}-|{C}_{r}(i,{t}_{1})-{C}_{r}(i,{t}_{2})|.$$

C_0_ captures the loss of coherence over time-periods shorter than the available Sentinel-1 repeat interval of 12 days (6 days if Sentinel-1a and -1b data are available), including the effects of surface roughness on coherence. Δ*t* is the timespan of individual interferograms, and *τ* describes the slope of the time-dependent loss of coherence. Here we model this decay as linear, although clearly that relationship does not hold at very long time intervals (coherence would become negative). Coherence in all interferograms between dates 6 months or longer after the rain event (presumably unbiased by coherence loss associated with soil moisture) decays approximately linearly with time, with a magnitude that is small compared with the variations in C_r_. We impose a negativity constraint on *τ*, such that coherence must decrease as the time-span between SAR acquisitions increases. This constraint does not significantly change the final result, but does keep the predicted coherence values from having non-physical values greater than one.

The *C*_*r*_(*i*, *t*_*j*_) term involves the changes in soil moisture that the affect strength of interaction between the radar signal and the scatterers within the shallow subsurface. Coherence between two dates decreases when the set of scatterers change following either an increase or decrease in soil moisture on one of the two dates. Since the effect is the same regardless of the sign of that increase or decrease (i.e. a wet first date vs. a dry second date gives the same change as a dry vs. wet pair), we use the absolute value of the difference between C_r_ on two dates, as in Equation 2. The inversion for C_r_ is nonlinear because of the absolute value term, and has an ambiguity in sign, and in terms of an absolute shift (i.e., you could add a constant to all values and get the same fit). We normalize the inversion by constraining the mean of values at dates 6 months or more after the rain event to be zero and requiring that the first post-event date have positive C_r_. This normalization does not affect the differences between C_r_ on individual dates. We use a trust-region reflective algorithm to perform the inversion, with multiple starting models, and find that it is robust to within the 0.1 coherence unit errors that we find for other aspects of our analysis. The inferred C_r_ is shown in Fig. 4 as a function of time at several pixels and all available tracks, with the fit to the full coherence dataset at one pixel and one track shown in Fig. 3 and Supplementary Figures 3–5.

We also infer a coherence loss (C_p_) that reflects permanent changes in the radar scatterers, likely associated with processes such as overland flow and debris transport during and shortly after the rain event. We define C_p_ as the difference between C_r_ averaged over pre-event dates and the C_r_ averaged over dates 6 months or later after the rain event. Because two pre-rain event SAR acquisitions are available for Track 54, this permanent change can be easily seen in Fig. 4b (black dots) where the values of C_r_ for the two pre-event dates are both negative, but very similar to each other – interferograms between those two dates have high coherence. The loss in coherence for the other two tracks on the single pre-event date relative to the rest of the time series could be assigned either to a permanent coherence loss or to a variation in soil moisture. However, the similarity to Track 54 (Fig. 5) and uniformly high coherence of dates 6 months after the rain event supports the idea that this loss of coherence was permanent and was likely related to permanent changes in the surface.

### Temporal decay rates

C_r_ decays to the background, near-zero values over a time period of >1 month at most pixels, and includes a signal from the secondary, smaller rain event in many locations (Fig. 4). To characterize the temporal decay of C_r_, we fit a piecewise exponential function to the inferred z_t_ values of the following form,

3 $$\begin{array}{rcl}{C}_{r}(i,t) & = & {A}_{1}{\rm{\exp }}(\frac{-t}{{\tau }_{1}})\,{\rm{for}}\,{\rm{March}}\,24,2015 < {\rm{t}} < {\rm{August}}\,8,2015\\ {C}_{r}(i,t) & = & {A}_{1}{\rm{\exp }}(\frac{-t}{{\tau }_{1}})+{A}_{2}{\rm{\exp }}(\frac{-t}{{\tau }_{2}})\,{\rm{for}}\,{\rm{t}} > {\rm{August}}\,8,2015\end{array}$$

where *τ*_1_ and *τ*_2_ describe the decay of the effect of the soil moisture associated with the March 24–26, 2015 and the August 8–9, 2015 rain events.

### Rainfall and atmospheric data

Rainfall (Figs 1 and 4) is reported at stations distributed throughout the research area^37^ and models of horizontal wind orientations^37, 38^ are consistent with the NNW-SSE orientation of streaks of lower correlation apparent in the C_p_ product (Fig. 5). Most of the precipitation data is available to the public at http://dgasatel.mop.cl/, via the Dirección General de Aguas (DGA), a Chilean governmental agency. Other sources are described in Jordan *et al*.^37^, Table S2.

## Electronic supplementary material

Supplemental Info

## References

[1] Rosen PA (2000). Synthetic Aperture Radar Interferometry. Proc IEEE.

[2] Burgmann R, Rosen PA, Fielding EJ (2000). Synthetic aperture radar interferometry to measure Earth’s surface topography and its deformation. Annu. Rev. Earth Planet. Sci..

[3] Massonnet D (1993). The displacement field of the Landers earthquake mapped by radar interferometry. Nature.

[4] Wdowinski S, Smith-Konter B, Bock Y, Sandwell D (2007). Diffuse interseismic deformation across the Pacific–North America plate boundary. Geology.

[5] Lindsey EO (2015). Line-of-sight displacement from ALOS-2 interferometry: *M*_*w*_ 7.8 Gorkha Earthquake and *M*_*w*_ 7.3 aftershock. Geophys. Res. Lett..

[6] Massonnet D, Holzer T, Vadon H (1997). Land subsidence caused by the East Mesa geothermal field, California, observed using SAR interferometry. Geophys. Res. Lett..

[7] Amelung F, Galloway DL, Bell JW, Zebker HA, Laczniak RJ (1999). Sensing the ups and downs of Las Vegas: InSAR reveals structural control of land subsidence and aquifer-system deformation. Geology.

[8] Yan Y (2012). Mexico City subsidence measured by InSAR time series: joint analysis using PS and SBAS approaches. Sel. Top. Appl. Earth Obs. Remote Sens. IEEE J. Of.

[9] Prush V, Lohman R (2014). Time-Varying Elevation Change at the Centralia Coal Mine in Centralia, Washington (USA), Constrained with InSAR, ASTER, and Optical Imagery. Sel. Top. Appl. Earth Obs. Remote Sens. IEEE J. Of.

[10] Prush V, Lohman R (2014). Forest Canopy Heights in the Pacific Northwest Based on InSAR Phase Discontinuities across Short Spatial Scales. Remote Sens..

[11] Handwerger AL, Roering JJ, Schmidt DA (2013). Controls on the seasonal deformation of slow-moving landslides. Earth Planet. Sci. Lett..

[12] Pritchard ME, Simons M (2004). An InSAR-based survey of volcanic deformation in the central Andes. Geochem. Geophys. Geosystems.

[13] Lundgren P (2004). Gravity and magma induced spreading of Mount Etna volcano revealed by satellite radar interferometry. Geophys. Res. Lett..

[14] Jung HS, Lu Z, Won JS, Poland MP, Miklius A (2011). Mapping Three-Dimensional Surface Deformation by Combining Multiple-Aperture Interferometry and Conventional Interferometry: Application to the June 2007 Eruption of Kilauea Volcano, Hawaii. IEEE Geosci. Remote Sens. Lett..

[15] Ebmeier SK (2012). Measuring large topographic change with InSAR: Lava thicknesses, extrusion rate and subsidence rate at Santiaguito volcano, Guatemala. Earth Planet. Sci. Lett..

[16] Zebker HA, Villasenor J (1992). Decorrelation in interferometric radar echoes. IEEE Trans. Geosci. Remote Sens..

[17] Dobson M, Ulaby F (1986). Active Microwave Soil Moisture Research. *IEEE Trans*. Geosci. Remote Sens..

[18] McCaulley JF (1982). Subsurface Valleys and Geoarcheology of the Eastern Sahara Revealed by Shuttle Radar. Science.

[19] Zebker HA, Weber Hoen E (2000). Penetration depths inferred from interferometric volume decorrelation observed over the Greenland Ice Sheet. IEEE Trans. Geosci. Remote Sens..

[20] Treuhaft RN, Madsen SN, Moghaddam M, van Zyl JJ (1996). Vegetation characteristics and underlying topography from interferometric radar. Radio Sci..

[21] Yun, S. *et al*. Damage Proxy Map of M6.3 Christchurch Earthquake Using InSAR Coherence. In Fringe 2011 Workshop: Advances in the Science and Applications of SAR Interferometry from ESA and 3rd Party Missions (2011).

[22] Yun S-H (2015). Rapid Damage Mapping for the 2015 Mw 7.8 Gorkha Earthquake Using Synthetic Aperture Radar Data from COSMO–SkyMed and ALOS-2 Satellites. Seismol. Res. Lett..

[23] Le Toan T, Beaudoin A, Riom J, Guyon D (1992). Relating forest biomass to SAR data. IEEE Trans. Geosci. Remote Sens..

[24] Sandberg G, Ulander LMH, Fransson JES, Holmgren J, Le Toan T (2011). L- and P-band backscatter intensity for biomass retrieval in hemiboreal forest. Remote Sens. Environ..

[25] Cloude SR, Papathanassiou KP (1998). Polarimetric SAR interferometry. Geosci. Remote Sens. IEEE Trans. On.

[26] Simard M (2012). An Empirical Assessment of Temporal Decorrelation Using the Uninhabited Aerial Vehicle Synthetic Aperture Radar over Forested Landscapes. Remote Sens.

[27] Wang JR, Shiue JC, Schmugge TJ, Engman ET (1989). Mapping surface soil moisture with L-band radiometric measurements. Remote Sens. Environ..

[28] Dubois PC, van Zyl J, Engman T (1995). Measuring soil moisture with imaging radars. IEEE Trans. Geosci. Remote Sens..

[29] Ulaby FT, Dubois PC, van Zyl J (1996). Radar mapping of surface soil moisture. J. Hydrol..

[30] Gabriel AK, Goldstein RM, Zebker HA (1989). Mapping small elevation changes over large areas: Differential radar interferometry. J. Geophys. Res. Solid Earth.

[31] Nolan M, Fatland DR (2003). Penetration depth as a DInSAR observable and proxy for soil moisture. IEEE Trans. Geosci. Remote Sens.

[32] Nolan M, Fatland DR, Hinzman L (2003). DInSAR measurement of soil moisture. IEEE Trans. Geosci. Remote Sens.

[33] Rabus B, Wehn H, Nolan M (2010). The Importance of Soil Moisture and Soil Structure for InSAR Phase and Backscatter, as Determined by FDTD Modeling. IEEE Trans. Geosci. Remote Sens.

[34] Ulaby F, Bradley G, Dobson M (1979). Microwave Backscatter Dependence on Surface Roughness, Soil Moisture, and Soil Texture: Part II-Vegetation-Covered Soil. IEEE Trans. Geosci. Electron..

[35] Barrett BW, Dwyer E, Whelan P (2009). Soil Moisture Retrieval from Active Spaceborne Microwave Observations: An Evaluation of Current Techniques. Remote Sens..

[36] De Zan F, Parizzi A, Prats-Iraola P, López-Dekker PA (2014). SAR Interferometric Model for Soil Moisture. IEEE Trans. Geosci. Remote Sens..

[37] Jordan, T. E. *et al*. The exceptional and extreme rain event of March 2015 in Northern Chile: Atmospheric conditions, hydrological results, and unanswered questions. *Submitt. Geophys Res Let* (2016).

[38] Jordan, T. E. *et al*. Hydrological and geomorphological consequences of the extreme precipitation event of 24–26 March 2015, Chile. In *XIV Congreso Geologico Chileno* (*La Serena*) (2015).

[39] Wilcox AC (2016). An integrated analysis of the March 2015 Atacama floods: The 2015 Atacama floods. Geophys. Res. Lett..

[40] Tapia, L. *et al*. Relación entre las distintas superficies y sus perfiles de humedad, post precipitaciones de marzo 2015, en la Sierra de Varas. In *II Región de Antofagasta - Chile*: *XIV Congreso Geológico Chileno* (2015).

[41] Bozkurt D, Rondanelli R, Garreaud R, Arriagada A (2016). Impact of Warmer Eastern Tropical Pacific SST on the March 2015 Atacama Floods. Mon Wea Rev.

[42] Dobson MC (1992). Dependence of radar backscatter on coniferous forest biomass. IEEE Trans. Geosci. Remote Sens.

[43] Sexton JO, Bax T, Siqueira P, Swenson JJ, Hensley S (2009). A comparison of lidar, radar, and field measurements of canopy height in pine and hardwood forests of southeastern North America. For. Ecol. Manag..

[44] Zwieback S, Hensley S, Hajnsek I (2015). Assessment of soil moisture effects on L-band radar interferometry. Remote Sens. Environ..

[45] Gamba P, Dell’Acqua F, Trianni G (2007). Rapid Damage Detection in the Bam Area Using Multitemporal SAR and Exploiting Ancillary Data. IEEE Trans. Geosci. Remote Sens..

[46] Plank S (2014). Rapid Damage Assessment by Means of Multi-Temporal SAR – A Comprehensive Review and Outlook to Sentinel-1. Remote Sens..

[47] Rosen, P. A., Gurrola, E., Sacco, G. F. & Zebker, H. The InSAR scientific computing environment. In *EUSAR 2012*; *9th European Conference on Synthetic Aperture Radar* 730–733 (2012).

[48] Luo X, Askne J, Smith G, Dammert P (2000). Coherence Characteristics of Radar Signals From Rough Soil - Abstract. J. Electromagn. Waves Appl..

[49] Joughin, L. R. & Winebrenner, D. P. Effective number of looks for a multilook interferometric phase distribution. In *Geoscience and Remote Sensing Symposium, 1994. IGARSS ’94. Surface and Atmospheric Remote Sensing*: *Technologies, Data Analysis and Interpretation. International* **4**, 2276–2278 vol. 4 (1994).

[50] Wessel P, Smith WHF, Scharroo R, Luis J, Wobbe F (2013). Generic Mapping Tools: Improved Version Released. Eos Trans. Am. Geophys. Union.

