# Supplementary material for: InSAR constraints on soil moisture evolution after the March 2015 extreme precipitation event in Chile
Source: Sci Rep. 2017 Jul 7;7:4903. doi: 10.1038/s41598-017-05123-4 (PMC5501829; doi:10.1038/s41598-017-05123-4)
Supplement: Supplementary file 1 — Supplemental Info [file 41598_2017_5123_MOESM1_ESM.doc]

Title: InSAR constraints on soil moisture evolution after the March 2015 extreme precipitation event in Chile (Supplementary Information)

C. P. Scott^a,1^, cps242@cornell.edu

R. B. Lohman^a^, rbl62@cornell.edu

T. E. Jordan^a^, tej1@cornell.edu

^a^Department of Earth and Atmospheric Sciences, Cornell University, Ithaca, NY 14853

^1^Present address: School of Earth and Space Exploration, Arizona State University, Tempe, AZ, 85287

Corresponding author: Rowena Lohman, rbl62@cornell.edu

| Date | Baseline (m) relative to March 9, 2015 | Ionosphere | Polarization |
| --- | --- | --- | --- |
| March 9, 2015 | 0 | No | VV |
| April 2, 2015 | 136 | No | VV |
| April 26, 2015 | 22 | No | VV |
| May 20, 2015 | 69 | No | VV+HV |
| June 13, 2015 | -15 | No | VV |
| July 7, 2015 | 89 | No | VV |
| August 24, 2015 | -34 | No | VV |
| September 17, 2015 | 79 | No | VV |
| October 11, 2015 | 66 | No | VV |
| November 4, 2015 | 19 | No | VV |
| November 28, 2015 | 49 | No | VV |
| January 15, 2016 | 43 | No | VV |
| March 3, 2016 | -7 | No | VV |
| April 8, 2016 | -9 | No | VV+HV |
| April 20, 2016 | 67 | No | VV |
| May 2, 2016 | 30 | No | VV+HV |
| June 7, 2016 | 59 | No | VV |
| July 1, 2016 | -8 | Yes | VV |
| July 25, 2016 | 31 | Yes | VV |
| August 18, 2016 | 60 | No | VV |

**Supplementary Table 1:** **Descending Track 156.**

Ionosphere denoted as “yes” indicates that the image is contaminated by ionospheric signals and that the associated SAR acquisition has been removed from the analysis. 20 dates available, 18 used in 153 interferograms.

| Date | Baseline (m) relative to January 1, 2015 | Ionosphere | Polarization |
| --- | --- | --- | --- |
| January 1, 2015 | 0 | No | VV |
| March 2, 2015 | 21 | No | VV |
| May 13, 2015 | 124 | No | VV+HV |
| June 30, 2015 | -66 | No | VV |
| July 24, 2015 | -84 | No | VV |
| August 17, 2015 | 39 | No | VV |
| September 10, 2015 | 20 | No | VV |
| October 4, 2015 | -47 | No | VV |
| October 28, 2015 | 120 | No | VV |
| November 21, 2015 | 8 | No | VV |
| December 15, 2015 | 11 | No | VV |
| January 8, 2016 | 89 | No | VV |
| February 25, 2016 | 31 | No | VV |
| March 8, 2016 | 50 | No | VV+HV |
| March 20, 2016 | 9 | No | VV |
| April 13, 2016 | -39 | No | VV |
| May 31, 2016 | 13 | No | VV |
| July 18, 2016 | -31 | No | VV |

**Supplementary Table 2: Descending Track 54.**

18 dates available and used in 153 interferograms.

| Date | Baseline (m) relative to March 13, 2015 | Ionosphere | Polarization |
| --- | --- | --- | --- |
| March 13, 2015 | 0 | No | VV |
| April 30, 2015 | 144 | No | VV |
| May 24, 2015 | 51 | No | VV |
| June 17, 2015 | 54 | No | VV |
| July 11, 2015 | -21 | No | VV |
| August 28, 2015 | 107 | No | VV |
| September 21, 2015 | 31 | No | VV |
| October 15, 2015 | 92 | No | VV |
| November 8, 2015 | 0 | No | VV |
| December 2, 2015 | 54 | No | VV |
| December 26, 2015 | 133 | No | VV |
| February 12, 2015 | 16 | No | VV |
| March 7, 2016 | 102 | No | VV |
| March 31, 2016 | 27 | No | VV |
| April 24, 2016 | 76 | No | VV |
| May 18, 2016 | 23 | No | VV |
| June 11, 2016 | 37 | No | VV |
| July 5, 2016 | 113 | Yes | VV |
| July 29, 2016 | 29 | No | VV |
| August 22, 2016 | 73 | No | VV |
| September 15, 2016 | 53 | No | VV |

**Supplementary Table 3: Ascending Track 47.**

21 dates available, and 20 used to make 190 interferograms.


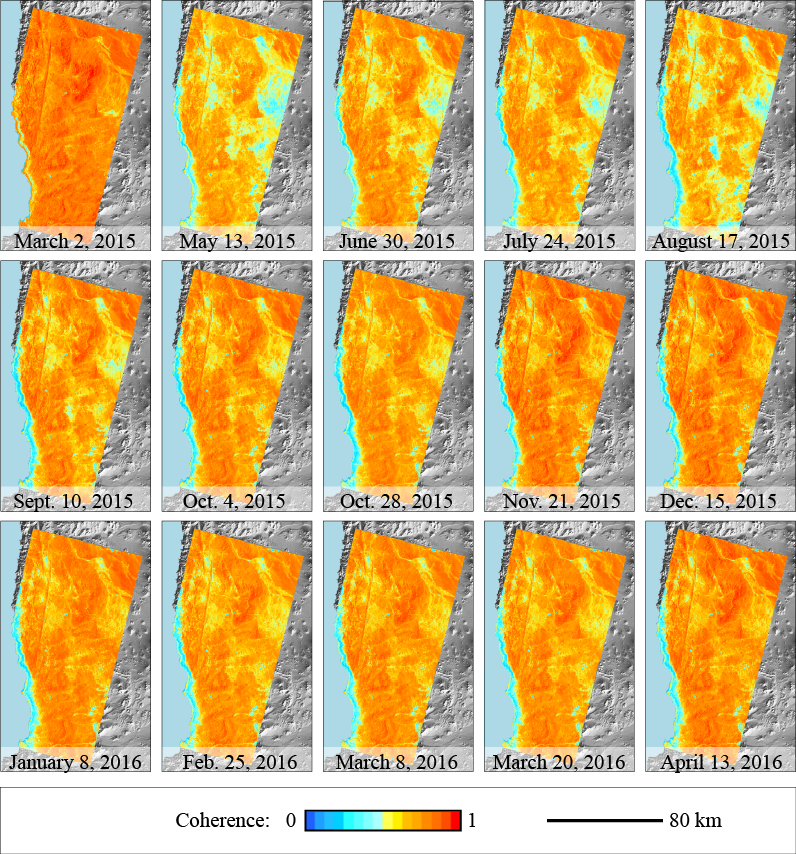


**Supplementary Figure 1: Coherence for Track 54, Swath 1.**

Coherence for interferograms between January 1, 2015, and all available dates through April, 2016. Note that interferograms between all pairs of dates were used in our analysis, not just those shown here. Figures generated using Generic Mapping Tools^50^, v. 5.2.1.

**
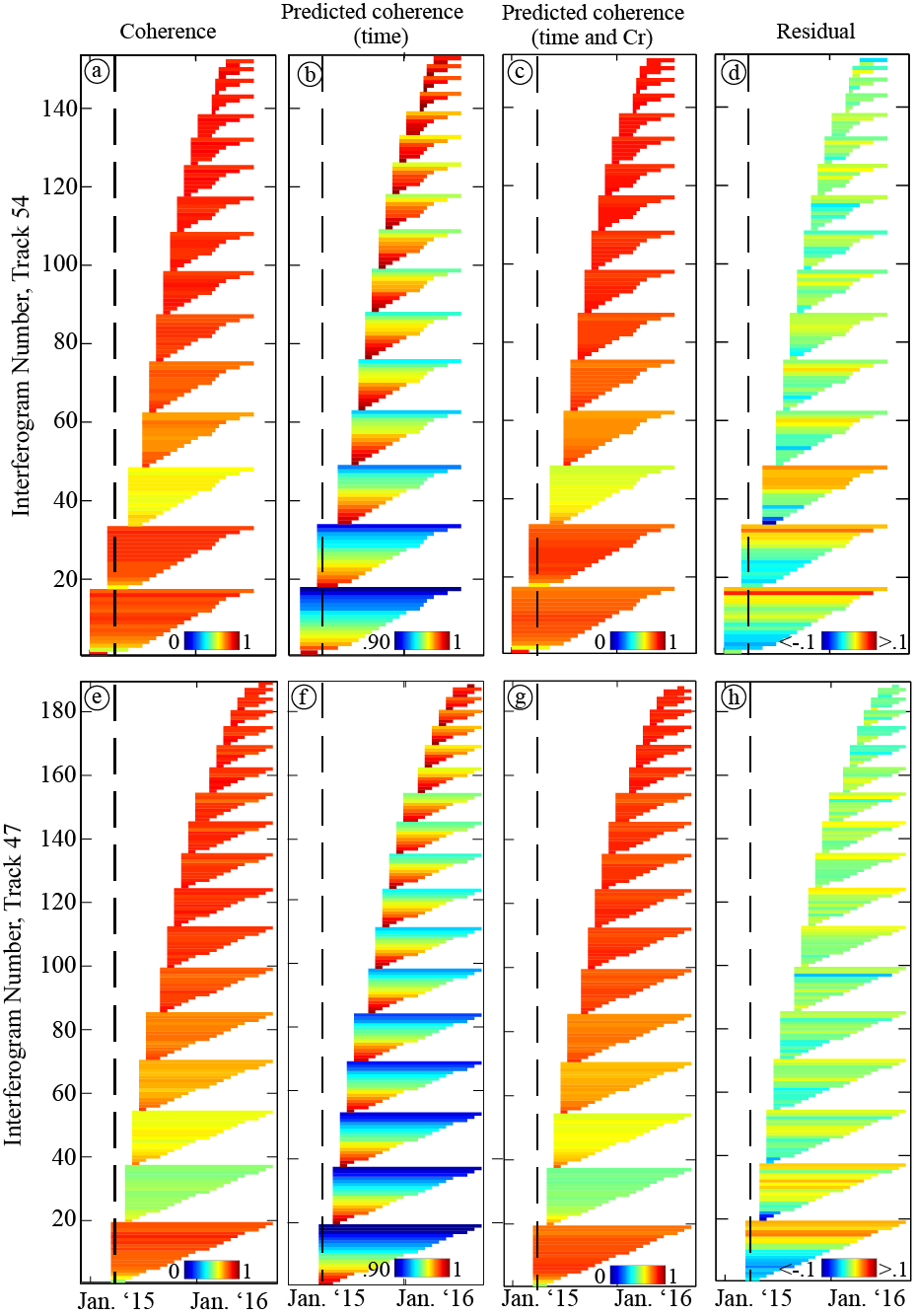
**

**Supplementary Figure 2: Coherence, all interferograms, all tracks.**

All data, shown as in Figure 3, for the other two tracks covering the same alluvial fan site, all on same color scales as in Figure 3. Note that Track 54 (top row) includes two dates before the rain event.

**
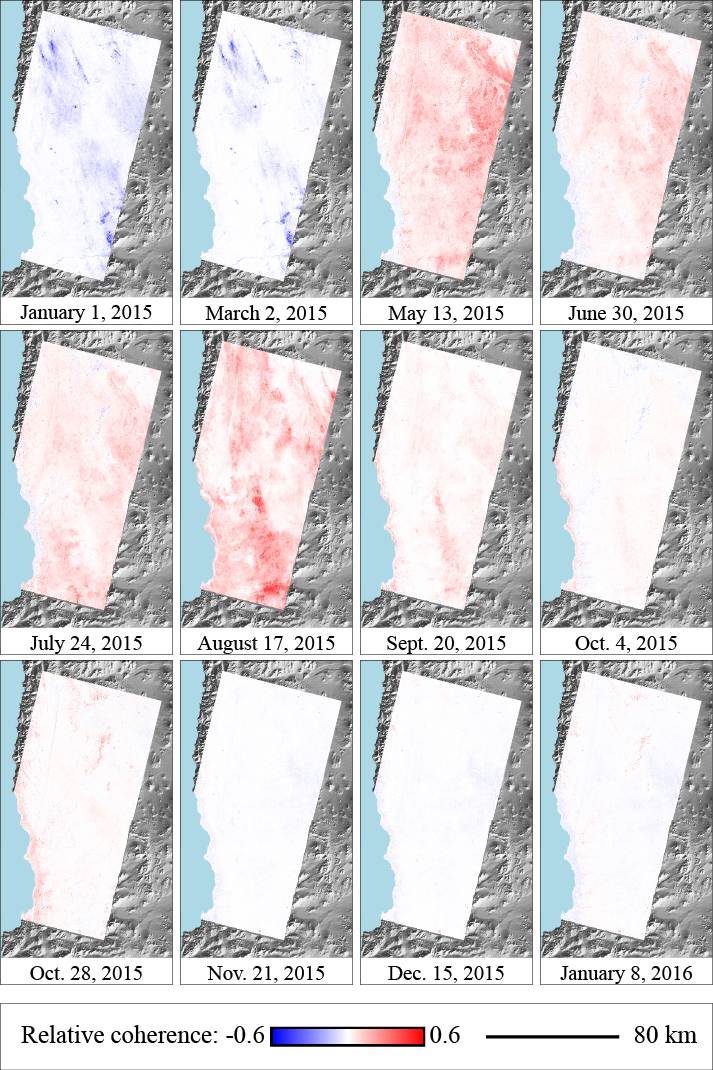
**

**Supplementary Figure 3: C_r_ for Track 54.**

Same as Supplementary Figure 1, but for maps inferred values of C_r_ for both swaths 1 and 2. Figures generated using Generic Mapping Tools^50^, v. 5.2.1.

**
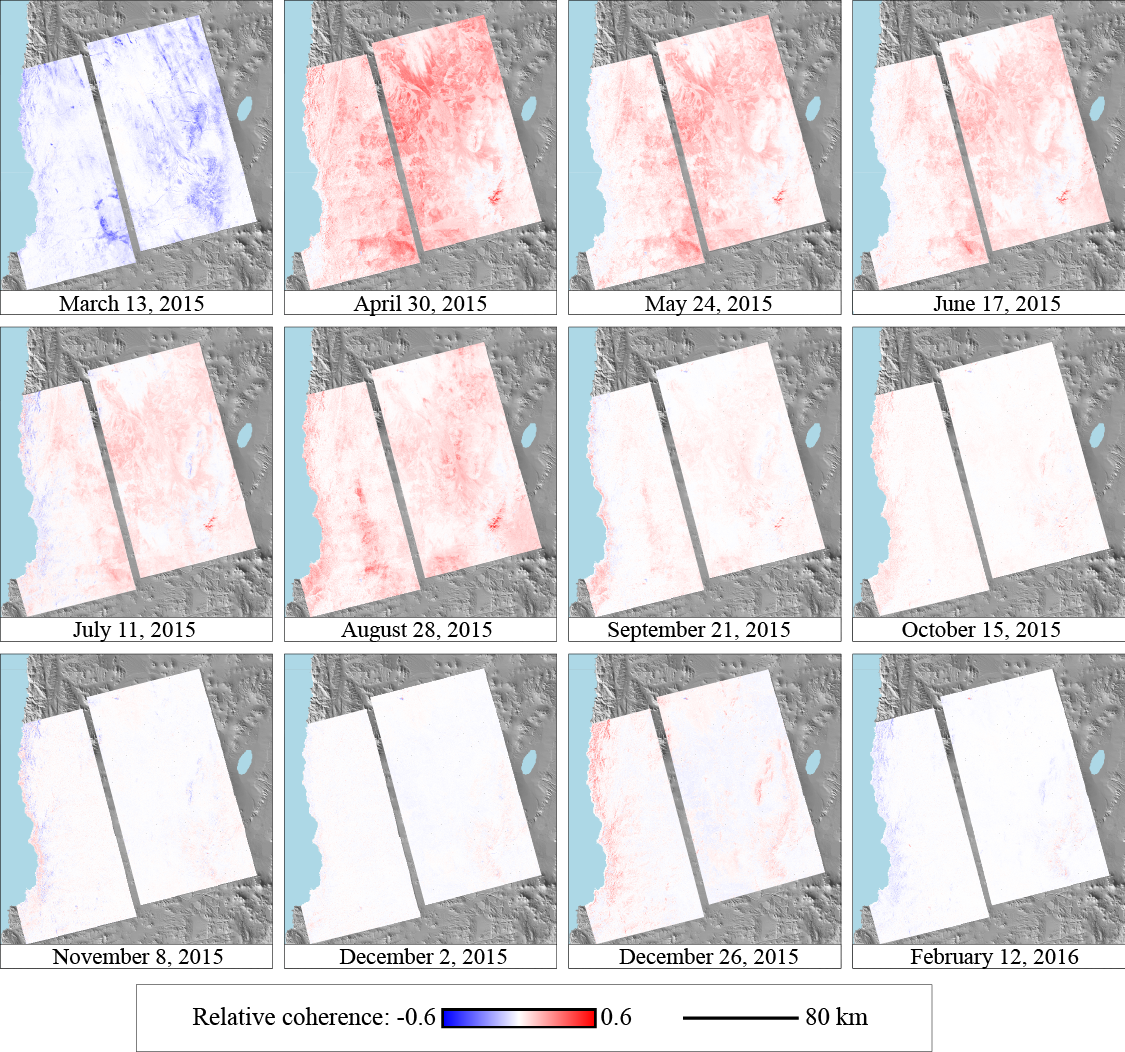
Supplementary Figure 4: C_r_ for Track 47.**

Same as Supplementary Figure 3, for Track 47 swaths 1 and 2. Figures generated using Generic Mapping Tools^50^, v. 5.2.1.

**
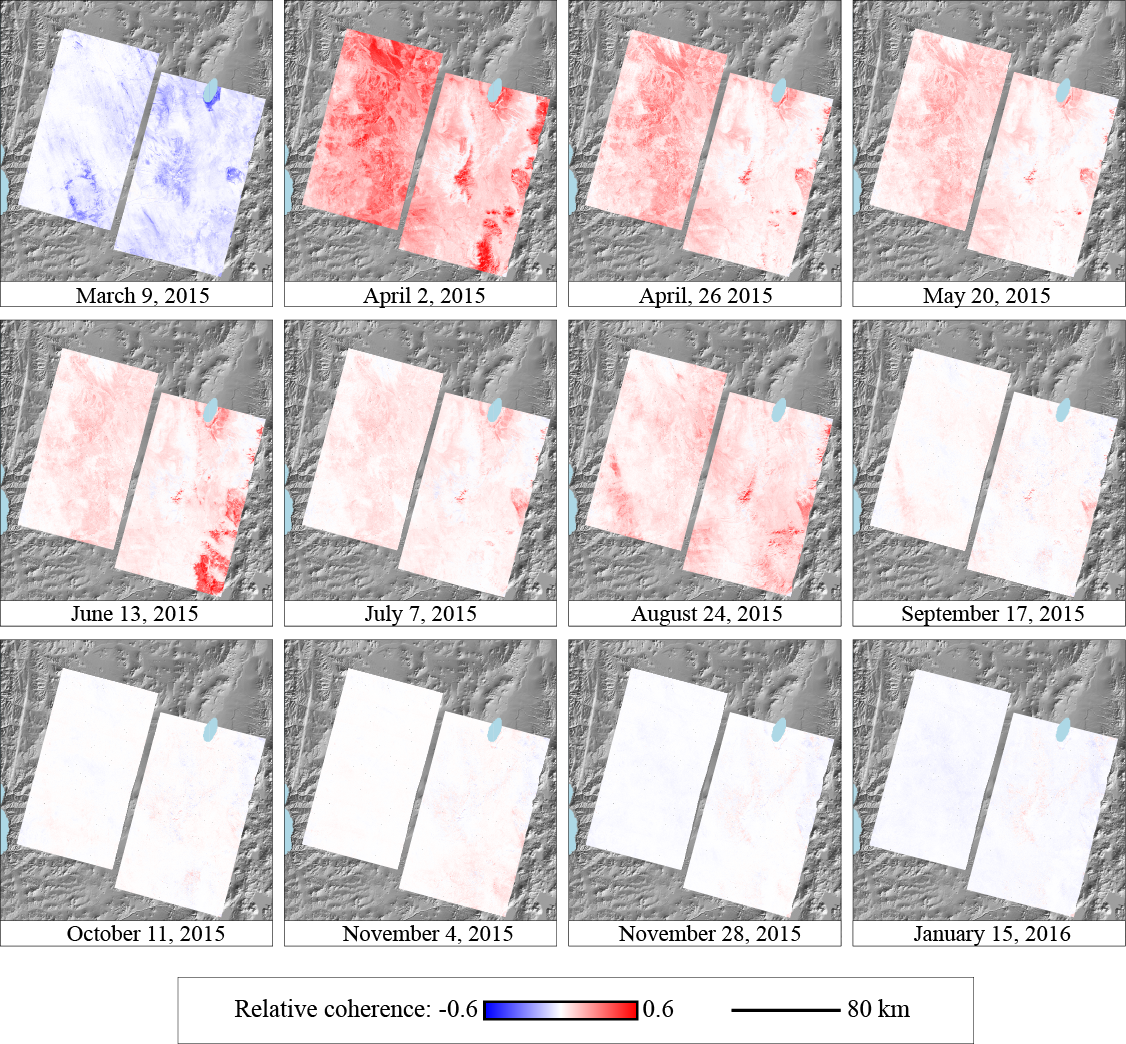
Supplementary Figure 5: C_r_ for Track 156.**

Same as Supplementary Figure 3, for Track 156, swaths 2 and 3. Figures generated using Generic Mapping Tools^50^, v. 5.2.1.


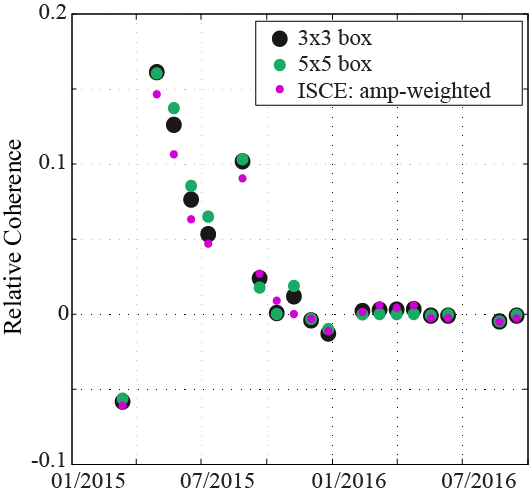


**Supplementary Figure 6: Dependence on phase variance method**

C_r_ computed for the same location, with input values of phase variance generated over a 3x3 and 5x5 window (unweighted by amplitude) and the results using ISCE (amplitude weighted).
